# Navigating the crossroads: exploring the intersection of celiac disease and Budd–Chiari syndrome – insights, challenges, and management strategies

**DOI:** 10.1097/MS9.0000000000003320

**Published:** 2025-04-22

**Authors:** Priyanka Mohan Lal, Heema Madhumal, Muhammad Hamza Siddiqui, Syed Shahrukh Parvez, Syed Shahzil Parvez, Mustafa Ashiq Husain, Komal Tirath, Anmol Mohan, Dev Tanush, Nikhil Duseja, Muhammad Khuzzaim Khan, Syeda Laiba Sherazi, Usha Tejwaney

**Affiliations:** a Ziauddin Medical University, Karachi, Pakistan; b Dow University of Health Sciences, Karachi, Pakistan; c Frimley Health NHS Foundation Trust, Frimley, UK; d Karachi Medical and Dental College, Karachi, Pakistan; e I.K. Akhunbaev Kyrgyz State Medical Academy, Bishkek, Kyrgyzstan; f Valley Health System, New Jersey, USA

## Abstract

Celiac disease (CD) and Budd–Chiari syndrome (BCS) are distinct medical conditions affecting different organ systems. However, reports suggest a potential association between them. This review examines existing literature and summarizes the current knowledge regarding the connection between CD and BCS. The pathophysiology of both conditions involves immune dysregulation and prothrombotic tendencies, though through different mechanisms. Several case reports and small studies indicate an increased incidence of BCS in individuals with CD, but the underlying mechanisms remain unclear. Proposed hypotheses include chronic inflammation, hypercoagulability, and potential genetic factors. However, more robust studies are needed to establish a definitive association and elucidate the shared pathophysiological factors between the two conditions. Management of CD primarily involves strict adherence to a gluten-free diet, leading to symptom improvement and normalization of serological markers. BCS requires a stepwise approach involving anticoagulation, endovascular therapy, and, in severe cases, liver transplantation. Recognizing the potential implications of the coexistence of CD and BCS is crucial for developing appropriate management strategies. Further research is warranted to investigate the potential association between CD and BCS, including large-scale epidemiological studies, genetic analyses, and mechanistic investigations. Understanding the underlying mechanisms and identifying risk factors for BCS development in individuals with CD will contribute to enhanced diagnostic capabilities, refined management strategies, and improved patient outcomes.

## Introduction

Celiac disease (CD) is a common immune-mediated disorder that affects the small intestine. It occurs in genetically predisposed individuals and is triggered by the ingestion of gluten, which is found in wheat, rye, and barley^[1]^. The intake of gluten and the presence of specific human leukocyte antigen (HLA) types are key factors in the development of CD. Extra-intestinal manifestations of CD include conditions such as type 1 diabetes mellitus and dermatitis herpetiformis. CD is also associated with various liver conditions, including nodular liver regeneration, autoimmune hepatitis, and silent transaminitis^[2]^. Recent studies have also suggested that alterations in the Gall bladder and bile tract can occur in CD patients. These changes can partially justify the observed enzymatic alterations in such individuals^[3]^. CD affects approximately 1% of the global population. The incidence of CD is rising worldwide, and the prevalence varies across different regions, which cannot be solely explained by recognized genetic and environmental risk factors^[4]^. First-degree relatives of individuals with CD, as well as those with type 1 diabetes or IgA deficiency, show a higher prevalence (10–15%) of the condition^[5]^.

**HIGHLIGHTS**

- The paper examines the link between celiac disease (CD) and Budd–Chiari syndrome (BCS), suggesting shared immune dysregulation and prothrombotic factors.
- It highlights a potential rise in BCS incidence among CD patients, possibly due to chronic inflammation and genetic predispositions, and calls for further research.
- The paper emphasizes a tailored management approach for patients with both CD and BCS, advocating a gluten-free diet for CD and a comprehensive treatment protocol for BCS, including anticoagulation and liver transplantation in severe cases.

Budd–Chiari syndrome (BCS) is a rare disorder characterized by thrombotic or non-thrombotic obstruction of the hepatic venous outflow tract, not associated with right-sided heart failure, sinusoidal obstruction syndrome, or constrictive pericarditis^[6]^. BCS is classified into primary and secondary types based on its etiology. Primary BCS occurs due to venous pathology (thrombosis or phlebitis), while secondary BCS results from external factors (such as a malignancy that compresses or invades the hepatic veins and/or inferior vena cava)^[7]^. Myeloproliferative disorders are the most common cause of BCS, with more than 80% of BCS patients having an underlying hypercoagulable condition^[8]^. A recent meta-analysis by Li et al. estimated the annual incidence of BCS to be between 0.168 and 4.09 per million, with subgroup analyses in Asian countries estimating an annual incidence of 0.469 per million^[9]^. Another survey indicated that BCS incidence is higher in men in Asian countries compared to women in non-Asian countries^[10]^. In comparison to adults, population-based studies on the epidemiology of BCS in children are missing^[11]^.

### Pathophysiology of BCS

BCS can be classified into primary (due to thrombosis or phlebitis of hepatic veins) and secondary (caused by external compression or invasion) types. About 75% of BCS cases have identifiable causes, while 25% remain idiopathic^[12]^. Primary BCS is strongly associated with thrombophilia, such as myeloproliferative disorders like polycythemia vera and essential thrombocythemia, often linked to JAK2 mutations^[13,14]^. Other prothrombotic factors include Factor V Leiden mutation (present in 25% of BCS cases), G20210A prothrombin gene mutation, and antiphospholipid syndrome^[15,16]^.

Secondary BCS results from malignancies (e.g., hepatocellular carcinoma) or traumas that compress or invade the hepatic venous outflow^[17,18]^. Hemodynamic changes in BCS involve obstruction of at least two hepatic veins, leading to sinusoidal congestion, portal hypertension, and hypoxic liver damage, which can progress to fibrosis and cirrhosis^[19,20]^. The caudate lobe is commonly affected due to its direct venous drainage into the inferior vena cava (IVC), causing its enlargement when hepatic veins are obstructed^[21]^.

### Pathophysiology of celiac disease

CD is an autoimmune enteropathy triggered by gluten in genetically predisposed individuals, involving both HLA (DQ2 and DQ8) and non-HLA genetic factors^[22,23]^. Gluten-derived peptides, such as gliadin, resist digestion, allowing them to traverse the intestinal epithelium. These peptides are deamidated by tissue transglutaminase (tTG), enhancing their binding to HLA-DQ2/8 molecules and activating CD4+ T cells, which drive inflammation through cytokine release (e.g., IFN-γ)^[24,25]^.

The resulting immune response causes villous atrophy, crypt hyperplasia, intraepithelial lymphocytosis, and hallmark features of CD. Additionally, innate immunity, primed by IL-15 and intraepithelial lymphocytes (IELs), contributes to mucosal damage^[26,27]^. Gluten-specific IEL activation further exacerbates tissue injury, but CD4+ T cells alone are insufficient to account for the epithelial destruction observed in CD^[28]^.

Differences in the histopathological changes between CD and BCS:

- CD: Villous atrophy, crypt hyperplasia, intraepithelial lymphocytosis, and lamina propria inflammation characterize intestinal biopsy findings^[29]^.
- BCS: Liver histopathology includes sinusoidal dilatation, centrilobular necrosis, perisinusoidal fibrosis, nodular regenerative hyperplasia, and parenchymal extinction due to prolonged ischemia^[30]^.

## Intestinal and extraintestinal manifestations of celiac disease

### Intestinal manifestations

CD presents with a range of gastrointestinal symptoms, often tied to malabsorption. In children, classic signs include chronic diarrhea, steatorrhea, abdominal pain, and failure to thrive, while severe cases may result in celiac crisis, characterized by diarrhea, hyponatremia, and shock^[31,32]^. Adults, however, more frequently present with nonspecific symptoms such as bloating, constipation, or irritable bowel syndrome-like complaints, including nausea and dyspepsia^[33]^.

### Extraintestinal manifestations

CD affects nearly all body systems, with extraintestinal symptoms occurring in about half of patients^[34]^. Common manifestations include iron deficiency anemia (40% of cases), osteoporosis (38–72%), and liver dysfunction, which may range from asymptomatic transaminase elevation to autoimmune liver disease^[35,36]^. Endocrine symptoms, such as type 1 diabetes and autoimmune thyroid disease, are also prevalent, with thyroid conditions affecting 10–15% of CD patients^[37]^.

Neurological complications, including peripheral neuropathy, cerebellar ataxia, and cognitive decline, often result from gluten sensitivity or vitamin deficiencies^[38,39]^. Gynecological issues such as infertility (OR 3.5, 95% CI 1.3–9) and adverse pregnancy outcomes, including recurrent miscarriages and low birth weight, are frequently observed^[40,41]^.

Dermatitis herpetiformis is a hallmark cutaneous manifestation, characterized by pruritic papules and vesicles on extensor surfaces. Other skin and oral conditions include psoriasis, urticaria, alopecia, and aphthous ulcers^[42]^. Rheumatic disorders, particularly juvenile idiopathic arthritis, are more common in CD patients, further highlighting its systemic nature^[43]^.

## Clinical presentation and diagnosis

### Budd–Chiari syndrome (BCS)

BCS can present variably, from sudden liver failure to asymptomatic cases. Most patients exhibit the classic triad of abdominal pain (61%), ascites (83%), and hepatomegaly (67%)^[44]^. Additional symptoms include fever, pedal edema, and, in severe cases, portal hypertension, variceal bleeding, or hepatic encephalopathy^[45,46]^. Children commonly present with rapidly reaccumulating ascites (83–90%) and dilated abdominal veins, with hepatomegaly often seen without ascites^[47]^.

Diagnosis relies on noninvasive imaging. Doppler ultrasonography, the first-line method (87.5% sensitivity), detects reversed blood flow, collateral circulation, and hepatic vein abnormalities, such as spider-web patterns or caudate lobe enlargement^[45,48]^. CT and MRI can confirm findings, show mottled liver perfusion, and identify extrahepatic manifestations like splenomegaly^[49,50]^. In small-vein obstruction, liver biopsy reveals congestion and fibrosis, while angiography identifies venous obstruction and collaterals^[51,52]^. Additional tests include coagulation studies, genetic testing for Factor V Leiden and prothrombin mutations, and bone marrow biopsy for myeloproliferative disorders^[53]^.

### Celiac disease

CD diagnosis involves serology and duodenal biopsy. IgA-tTG antibodies are the first-line test due to high sensitivity, while anti-endomysium (EmA) and deamidated gliadin peptide (DGP) antibodies provide supplementary accuracy^[54]^. Gluten consumption (3–10 g/day) is necessary for testing^[55]^. A duodenal biopsy remains the gold standard for adults, showing villous atrophy, crypt hyperplasia, and increased intraepithelial lymphocytes^[56,57]^.

In children, high anti-tTG titers (>10 × cutoff) combined with EmA and HLA-DQ2/DQ8 positivity may eliminate the need for biopsy in some cases, per ESPGHAN guidelines^[58]^. For children under 2 years, combining DGP-IgA/IgG with tTG-IgA improves accuracy^[59]^. HLA-DQ2/DQ8 testing is helpful but nonspecific, as these markers are present in 40% of the general population^[60]^.

### Association between celiac disease and Budd–Chiari syndrome

**Figure 1.**
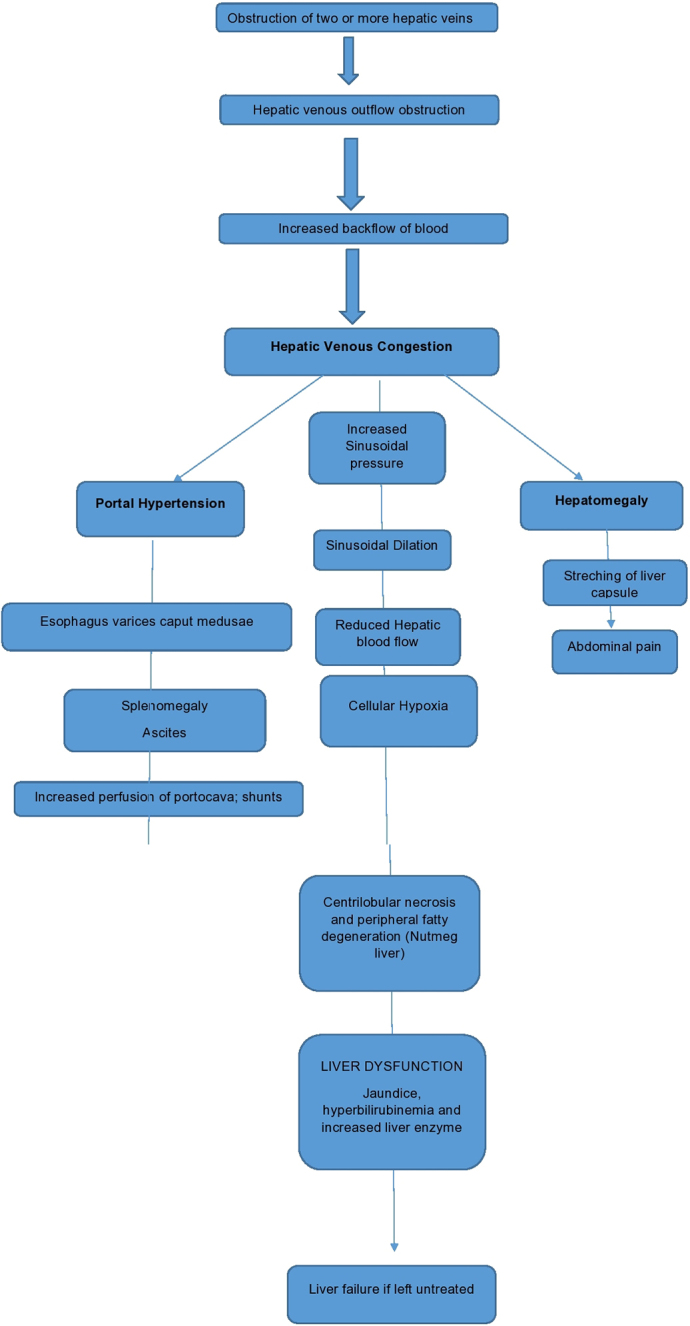
Pathophysiology of Budd–Chiari syndrome.

The association between CD and BCS was first documented in 1990^[61]^, and numerous reports since then highlighted this rare yet clinically significant link^[62]^. Most documented cases originate from North Africa, the Middle East, and southern Europe, regions where both conditions are more prevalent^[63-65]^ (Fig. 1).

### Case reports highlighting the association

Several case studies illustrate the clinical presentation of coexisting CD and BCS, providing valuable insights into this rare association. In Pakistan, Choudhry et al. reported a 25-year-old female with a three-year history of CD who developed BCS without any identifiable prothrombotic state^[66]^. Similarly, Ismail et al. described a 15-year-old girl with CD who adhered to a gluten-free diet for 6 years but still developed BCS despite having a normal thrombophilia profile^[67]^.

From India, Kochhar et al. documented a 19-year-old male with concurrent CD and BCS who was found to have deficiencies in protein C and protein S^[68]^. Another case from India by Meena et al. involved an 18-year-old female with a six-year history of CD presenting with subacute BCS, which was linked to elevated homocysteine levels and decreased folate levels^[69]^.

In Turkey, DANALIOĞLU et al. described a 24-year-old female with refractory ascites secondary to CD-associated BCS. Imaging revealed an enlarged caudate lobe and absent hepatic vein blood flow, while her thrombophilic profile was normal^[70]^. Additionally, Temani et al. reported a 5-year-old boy diagnosed with CD at age 2 who later developed BCS, presenting with failure to thrive, ascites, and hepatomegaly. Ultrasound findings included caudate lobe hypertrophy and narrowed hepatic vein lumen with collateral formations^[71]^. These cases collectively underscore the variability in clinical presentation and the need for heightened clinical suspicion for BCS in patients with CD, especially in regions where both conditions are common.

## Proposed mechanisms for the association

Several mechanisms have been proposed to explain the potential connection between CD and BCS:

- Ethnic and environmental factors: The higher prevalence of CD and BCS in regions such as North Africa may be influenced by shared genetic predispositions or environmental exposures. For instance, dietary hepatotoxins in the North African diet may exacerbate intestinal permeability, increasing thrombotic risks^[72]^. However, it remains unclear whether the perceived association is due to true causality or regional prevalence of both conditions^[73]^.
- Thrombophilic and hemostatic changes in CD: Approximately 61% of CD-associated BCS cases lack a specific thrombotic etiology, compared to over 80% of isolated BCS cases being linked to thrombophilic disorders^[74]^. This suggests CD itself may contribute to the hypercoagulable state via:
- Hyposplenism: Leads to thrombocytosis, increasing the risk of clot formation.
- Vitamin K malabsorption: Results in lower levels of protein C and protein S, impairing anticoagulation mechanisms.
- Hyperhomocysteinemia: Often due to MTHFR gene mutation or folate deficiency, amplifies thrombotic risks^[75,76]^.
- Autoimmune and inflammatory pathways: Autoimmune conditions associated with CD, such as lupus anticoagulant and vasculitis, may further enhance thrombotic risks. Secondary autoimmunity in progressive CD could explain the increased frequency of vascular events, including hepatic vein thrombosis^[77,78]^. Additionally, chronic inflammation in CD leads to elevated proinflammatory cytokines that disrupt endothelial function, increasing susceptibility to thrombosis.
- Nutritional deficiencies: Magnesium deficiency, commonly observed in CD, has been linked to thrombosis, including splenic vein thrombosis, highlighting the systemic impact of malabsorption in these patients^[79]^. Persistent deficiencies in key nutrients like folate, vitamin B12, and vitamin K may exacerbate the prothrombotic state, particularly in untreated or refractory CD.

### Clinical implications

The hypercoagulable state associated with CD may predispose patients to thrombotic complications in various venous systems, including hepatic, mesenteric, or spleno-portal veins, ultimately leading to diverse manifestations of BCS^[80]^. These observations emphasize the need for a multidisciplinary approach in managing CD patients who present with liver dysfunction or thrombotic events.

## Management strategies for Budd–Chiari syndrome (BCS) and celiac disease

*Budd–Chiari syndrome (BCS)*

1. Anticoagulation: The first-line treatment for BCS, anticoagulants prevent thrombosis progression and manage prothrombotic conditions. Low-molecular-weight heparin is preferred short-term, with long-term therapy using warfarin or vitamin K antagonists to maintain an INR of 2–3. Anticoagulation is also essential during procedures like stenting or liver transplantation to prevent complications^[81-84]^.
2. Endovascular therapy: Angioplasty and stenting are used to restore hepatic blood flow, with wall stents providing better long-term patency (80–90%) than balloon angioplasty alone. Local thrombolysis may also be considered for clot resolution^[85-87]^.
3. Transjugular intrahepatic portosystemic shunt (TIPS): TIPS is recommended for patients who fail angioplasty or thrombolysis. It prevents recurrent stenosis and reduces portal hypertension. Stent grafts or ePTFE-coated stents improve long-term outcomes compared to bare stents^[88-90]^.
4. Orthotopic liver transplantation: A last-resort option for severe cases with cirrhosis or liver failure when other therapies fail. Lifelong immunosuppression and anticoagulation are necessary post-transplant^[91,92]^.

*Celiac disease*

1. Gluten-free diet (GFD): A strict GFD is the cornerstone of CD treatment, improving symptoms and normalizing serological markers and intestinal histology. The FDA limits gluten in “gluten-free” foods to <20 ppm, though stricter avoidance may be required for some patients^[93]^.
2. Anticoagulation: In patients with CD-related thrombosis, anticoagulants like vitamin K antagonists are effective, with studies showing a 100% survival rate in treated individuals. Prophylactic anticoagulation in CD patients with elevated thrombotic risk requires further research^[94]^.
3. Impact of GFD on thrombosis: GFD reduces hypercoagulability by normalizing protein C, protein S, and homocysteine levels. It also alleviates secondary autoimmunity and reduces vascular complications. Studies show GFD compliance resolves ascites and reduces thrombosis in BCS-CD patients, while non-compliance leads to refractory symptoms and thrombosis progression^[95]^.

## Prognosis and complications

### Celiac disease (CD)

Late diagnosis (beyond age 50) or failure to adhere to a GFD increases mortality in CD. Complications include hyposplenism, refractory CD, intestinal lymphoma, small bowel cancer, and ulcerative jejunoileitis^[96]^. CD patients face an elevated risk of ischemic stroke, myocardial infarction, and venous thromboembolism (VTE), with a meta-analysis reporting a 25% higher VTE risk^[97]^. Ludvigsson et al. confirmed increased VTE risk in adults with CD, irrespective of gender^[98]^.

### Budd–Chiari syndrome (BCS)

BCS prognosis has improved due to early diagnosis and advances in treatments like anticoagulation. Survival rates increased from 50% (pre-1985) to 75% at 5 years. Interventional procedures to restore venous drainage yield the best outcomes, regardless of prognostic indices like MELD or BCS–TIPS scores^[99]^. Mortality is highest within 2 years of diagnosis and unrelated to therapy, with concerns including hepatocellular carcinoma and progression of underlying blood diseases^[100]^.

### Celiac disease and Budd–Chiari syndrome

Adherence to a GFD significantly improves prognosis for CD and reduces complications like intestinal villous atrophy and enteropathy-associated T-cell lymphoma (EATL)^[101]^. Refractory CD, defined as persistent symptoms despite GFD adherence, is linked to severe outcomes like lymphoma in rare cases^[102]^. CD patients also exhibit increased risks of thromboembolism and abnormal liver function tests, which typically resolve with a GFD^[103,104]^. For patients with both CD and BCS, outcomes improve with GFD and anticoagulation therapy, such as vitamin K antagonists^[104]^ (Table 1).

**Table 1:** Comparison between celiac disease and Budd–Chiari syndrome

Parameter	Celiac disease	Budd–Chiari syndrome
Clinical manifestation	Diarrhea, weight loss, abdominal pain, dermatitis, herpetiformis, iron deficiency anemia, osteoporosis, neurological symptoms	Sudden liver failure, abdominal pain, hepatomegaly, ascites, fever, pedal edema, portal hypertension leading to variceal bleeding and hepatic encephalopathy
Pathophysiology	Gluten induced damage to intestinal mucosa, villous atrophy, crypt hyperplasia, intraepithelial lymphocyte infiltration	Hepatic venous outflow obstruction, liver congestion, centrilobular hepatocyte hypoxia, fibrosis and cirrhosis
Differential diagnosis	Irritable bowel syndrome, inflammatory bowel disease, lactose intolerance, Small bacterial overgrowth	Hepatitis, cirrhosis, portal vein thrombosis, liver cancer
Diagnostic procedures	Serological tests (anti-tTG, anti-EMA), duodenal biopsy(standard), genetic testing for HLA-D2Q/DQ8	Imaging procedures (ultrasound, CT-scan, MRI), hepatic venography, liver biopsy
Hematological/biochemical abnormalities	Elevated anti-tTG antibodies, iron deficiency, vitamin deficiency	Elevated liver enzymes, coagulation abnormalities, thrombocytopenia, elevated bilirubin
Tissue biopsy programs (for diagnosis)	Duodenal biopsy to assess for villous atrophy and inflammation	Liver biopsy to evaluate for liver damage and cirrhosis
Treatment/management	Gluten free diet (GFD), nutritional supplementation, testing for other associated conditions	Anti-coagulation therapy, endovascular procedure (stenting, angioplasty), transjugular intrahepatic portosystemic shunt (TIPS), orthotropic liver transplantation
Educational activities	Dietary education, regular follow ups for adherence to GFD, awareness of associated conditions	Awareness and monitoring of underlying prothrombic conditions, management of complications and follow-ups after intervention

## Treatment challenges and considerations

### Budd–Chiari syndrome (BCS)

BCS involves partial or complete hepatic venous outflow obstruction and varies globally, with acute cases being more common in the West. Diagnostic and therapeutic challenges, especially in low-resource settings, include underrecognition and difficulty detecting small hepatic vein occlusions^[105]^. Restoring venous outflow post-liver transplantation often requires multiple procedures, with complications such as biliary issues and vascular thrombosis^[106]^.

Liver transplantation is reserved for fulminant liver failure, cirrhosis, or failed interventions, but there is debate regarding whether outcomes differ between BCS and non-BCS patients^[107,108]^. Early evidence-based management, including ultrasound and clinical suspicion, is critical to improving prognosis^[109]^ (Table 2).

**Table 2:** Current evidence of association between celiac disease and Budd–Chiari syndrome

Study	Location	Study design	Patient population	Findings	Relevance to CD-BCS association
Choudhry *et al* (1990)	Pakistan	Case report	1 female, 25 years old	Developed BCS with no identifiable prothrombotic state; CD diagnosed through biopsy.	First documented case of BCS in a CD patient without clear prothrombotic markers.
Ismail *et al* (1990)	Pakistan	Case report	1 female, 15 years old	Developed BCS despite a normal thrombophilia profile and adherence to a gluten-free diet.	Suggests that CD may be a risk factor for BCS despite normal thrombotic tests.
Kochhar *et al* (2000)	India	Case series	5 patients with CD and BCS	Patients had CD with deficiencies in Protein C and S; BCS found in conjunction with these deficiencies.	Links BCS with CD and specific thrombotic deficiencies.
Meena *et al* (2000)	India	Case report	1 female, 18 years old	BCS with elevated homocysteine and decreased folate levels; CD diagnosed with duodenal biopsy.	Highlights potential role of nutritional deficiencies in BCS development in CD patients.
Danalioğlu *et al* (2000)	Turkey	Case report	1 female, 24 years old	BCS with normal thrombophilic profile; confirmed CD with biopsy.	Indicates that BCS can occur in CD patients with normal thrombotic profiles.
Temani *et al* (2016)	Unknown	Case report	1 male, 5 years old	Developed BCS; imaging showed specific liver changes and ascites.	Demonstrates BCS development in a young CD patient.
General literature	Various	Literature review	Various CD and BCS cases	Up to 61% of CD-associated BCS cases have no identifiable thrombotic etiology; varying prevalence by region.	Suggests that CD may contribute to BCS development possibly due to unidentified thrombotic mechanisms.

### Celiac disease (CD)

A lifelong GFD is the only effective treatment, improving symptoms, quality of life, and reducing risks of complications like refractory CD and intestinal malignancies^[110]^. Persistent symptoms despite GFD adherence necessitate reevaluation of diagnosis, diet, or coexisting conditions^[111]^.

While effective, a GFD can lead to nutritional imbalances, including deficiencies in iron, calcium, magnesium, vitamins D, E, and B-complex^[112,113]^. Healing of the gut mucosa typically resolves these deficiencies within the first year, but long-term monitoring is vital. Educational programs on nutrition and the role of dietitians are essential to ensure balanced diets and adherence^[114,115,116]^.

### Future directions and research gaps

Advances in BCS management have raised 5-year survival rates to over 80%^[8]^. Further studies on personalized treatment, new anticoagulants, and TIPS efficacy in specific populations, such as Asian patients with IVC involvement, are needed^[117]^.

For CD, genetic and environmental factors are increasingly recognized as contributors. Genome-wide analyses have identified additional immune-related loci, supporting the hygiene hypothesis in autoimmune diseases^[104]^. Promising therapeutic options, such as enzyme-based gluten modification, may soon complement GFDs^[118]^.

Development of clinical tools for monitoring remission and managing screened cases is crucial, particularly for adolescents, where health perceptions and social impacts affect quality of life^[119]^.

## Conclusion

BCS and CD present distinct management challenges but also exhibit intriguing associations. BCS management involves a stepwise approach, starting with anticoagulants to prevent thrombosis, followed by endovascular treatments such as angioplasty and stenting. When these methods fail, TIPS and liver transplantation may be necessary. Despite advancements in treatment, further research is needed to understand the long-term outcomes and effectiveness of various prognostic indices.

CD, managed primarily through a strict GFD, resolves symptoms and normalizes serological markers. However, complications like enteropathy-associated T-cell lymphoma and other malignancies may occur in refractory cases. Anticoagulant therapy is recommended for CD patients with thrombosis, although its benefits require further study.

The potential link between BCS and CD warrants attention, as some studies suggest shared pathophysiological factors or increased risk of thrombotic events in patients with CD. Understanding this association could improve diagnostic and treatment strategies for both conditions. Future research should explore the impact of GFD on BCS and assess the role of anticoagulants in CD, aiming to enhance patient outcomes and quality of life. By addressing these knowledge gaps, we can better manage these complex conditions and their potential connections.

## Data Availability

The data are publicly available.
